# The Effect of *Lactobacillus plantarum* CQPC02 on Fatigue and Biochemical Oxidation Levels in a Mouse Model of Physical Exhaustion

**DOI:** 10.3389/fnut.2021.641544

**Published:** 2021-05-20

**Authors:** Ruokun Yi, Min Feng, Qiuping Chen, Xingyao Long, Kun-Young Park, Xin Zhao

**Affiliations:** ^1^Chongqing Collaborative Innovation Center for Functional Food, Chongqing University of Education, Chongqing, China; ^2^Department of Obstetrics, Eastern Hospital, Sichuan Provincial Medical Sciences Academy and Sichuan Provincial People's Hospital, Chengdu, China; ^3^Department of Education, Our Lady of Fatima University, Valenzuela, Philippines; ^4^Department of Food Science and Biotechnology, Cha University, Seongnam, South Korea

**Keywords:** *Lactobacillus plantarum* CQPC02, oxidation, physical exhaustion, mouse, swimming

## Abstract

Chinese Sichuan pickle is a fermented food rich in microorganisms. Microorganisms have the potential to become an important new form of potent future therapeutic capable of treating human disease. Selecting vitamin C as a positive control, a lactic acid bacteria (*Lactobacillus plantarum* CQPC02, LP-CQPC02) isolated from Sichuan pickle was given to mice over 4 weeks to investigate the effect of CQPC02 on fatigue levels and biochemical oxidation phenomena in exercise-exhausted Institute of Cancer Research (ICR) mice. The fatigue model was established by forced swimming of mice, the levels of hepatic glycogen, skeletal muscle glycogen, lactic acid, blood urea nitrogen and free fatty acid were measured by physicochemical methods, serum serum creatine kinase (CK), aspartate aminotransferase (AST) and alanine aminotransferase (ALT), superoxide dismutase (SOD), catalase (CAT) and malondialdehyde (MDA) levels were measured by kits, the histopathological changes in the livers of mice were observed by H&E slicing, and the mRNA changes in the livers and skeletal muscles were observed by quantitative polymerase chain reaction (qPCR). Both vitamin C and LP-CQPC02 increased swimming exhaustion time. The concentration of LP-CQPC02 and exhaustion time were positively correlated. LP-CQPC02 also increased liver glycogen, skeletal muscle glycogen and free fatty acid content in mice and reduced lactic acid and blood urea nitrogen content in a dose-dependent manner. As walnut albumin antioxidant peptide concentration increased, levels of mouse CK, AST, and AST gradually decreased. LP-CQPC02 increased SOD and CAT levels and decreased MDA levels in a dose-dependent fashion. LP-CQPC02 up-regulated expression of mRNA encoding copper/zinc-superoxide dismutase (Cu/Zn-SOD), manganese-superoxide dismutase (Mn-SOD), and CAT in swimming exhaustion mouse liver tissue. LP-CQPC02 also up-regulated alanine/serine/cysteine/threonine transporter 1 (ASCT1) expression while down-regulating syncytin-1, inducible nitric oxide synthase (iNOS), tumor necrosis factor-alpha (TNF-α) expression in swimming exhaustion mouse skeletal muscle. Overall, LP-CQPC02 had a clear anti-fatigue and anti-oxidation effect. This suggests that LP-CQPC02 can be developed as a microbiological therapeutic agent.

## Introduction

Traditional Chinese fermented food Sichuan pickle has a history stretching back over 2000 years. It is produced by first soaking and then fermenting fresh vegetables (1). Sichuan pickle is rich in active lactic acid bacteria due to the unique fermentation processes used in its production. Modern scientific methods have indicated that lactic acid bacteria and its metabolites, including lactic acid, account for 0.3–1.0% of Sichuan pickles (2). Studies have also shown that lactic acid bacteria in Sichuan pickles can promote the absorption of nutrients, improve intestinal function, reduce serum cholesterol level, and blood lipid concentration, and may also have anti hypertension and tumor effects able to regulate immune function and moderate diabetes (3–5). Korean kimchi, which is produced from the same fermented vegetables, has similar beneficial properties including decreasing food allergy, managing weight, and improving osteoporosis (6, 7). Therefore, lactic acid bacteria in Sichuan pickles have the potential to become a useful nutritional supplement or therapeutic microbial agent.

Fatigue is a physiological phenomenon caused by the body's inability to maintain the intensity of exercise (8). However, exercise-induced fatigue is common among athletes, students, those undertaking urban fitness, and diverse other groups. People recovering from illness and those in poor physical condition should receive regular exercise but can become prone to fatigue (9). Exercise fatigue is mainly generated by the rapid depletion of stored energy reserves caused by maintaining high-intensity exercise or exercising when the body is in poor condition, resulting in a transition to anaerobic energy metabolism and the excessive accumulation of metabolites related to fatigue (10). Excessive or strenuous exercise leads to cells moving from aerobic to anaerobic energy supply, causing the large accumulation of lactic acid, an imbalance in pH and osmotic pressure, and increased levels of reactive oxygen species (ROS). These are important physiological factors leading to fatigue (11). Moreover, ROS damages cell membrane integrity and induces oxidative skeletal muscle fatigue through lipid peroxidation: both are important factors leading to fatigue in the body, so effective scavenging of free radicals is key to alleviating fatigue (12).

Studies have found that lactic acid bacteria can act as probiotics (13). Other studies have shown some lactic acid bacteria enhance biological activity, detoxification, and immunity, act as adjuvant treatments of disease, and can thus be used as microbial drug moieties (14, 15). Because lactic acid bacteria isolated from food generally have high *de facto* safety, probably greater than most synthetic or biologic drugs, they should have greater prospects for use in the elderly, athletes, recuperating patients, and diverse other special or particularly sensitive populations.

The effect of lactic acid bacteria on exercise fatigue has not been well-studied, and the potential mechanisms-of-action remain unclear. This study investigated the effect of a recently-isolated lactic acid bacteria (LP-CQPC02) on fatigue and biochemical oxidation processes in an animal model of exhaustion, in order to provide an underpinning theoretical basis for the future development of exercise-orientated nutritional supplements or microbial-based pharmaceuticals.

## Materials and Methods

### Strain of LP-CQPC02

LP-CQPC02 is a lactic acid bacteria isolated from pickles naturally fermented in Chongqing, China. It was identified as *Lactobacillus plantarum* using 16S rRNA gene sequencing and named *Lactobacillus plantarum* CQPC02. The patent strain was preserved at the China General Microbiological Culture Collection Center, Beijing, China. Culture preservation number: CGMCC No. 14491.

### Animal Grouping and Handling

Seventy-five ICR mice (Experimental Animal Center of Chongqing Medical University, Chongqing, China) were randomly allotted to 5 groups: control, swimming, vitamin C, low-dose LP-CQPC02 (LP-CQPC02L), and high-dose LP-CQPC02 (LP-CQPC02H), with 15 mice per group. Mice from the vitamin C group were administered intragastrically with a solution of vitamin C at a dose of 100 mg/kg per day. Mice from the LP-CQPC02L and LP-CQPC02H groups were administered LP-CQPC02 intragastrically at respective doses of 1.0 × 10^8^ and 1.0 × 10^9^ CFU/kg. Mice from the control and swimming groups were administered daily 0.2 mL saline intragastrically. The five groups received intragastric administration for 4 weeks continuously. The protocol for these experiments was approved by the Ethics Committee of Chongqing Collaborative Innovation Center for Functional Food (202005022B), Chongqing, China.

### Swimming Exhaustion Experiments

After intragastric administration of vitamin C and LP-CQPC02, mice in the four non-control groups (swimming, vitamin C, LP-CQPC02L, and LP-CQPC02H) performed three sets of 30-min swimming exercises during the first week (KW-QP forced swimming system in mice, Nanjing Calvin Biotechnology Co., Ltd, Nanjing, Jiangsu, China). The mice were placed in the forced swimming system and allowed to swim freely. When the mice were powerless and floated freely in the water, after 15 s of rest, the water was stirred by a glass rod to urge the mice to continue swimming for 30 min until the mice completely exhausted and sank, and the mice were taken out to stop moving. Subsequently, swim time was reduced to 10 min, three times per week. In 4 weeks, after the final LP-CQPC02H gavage, mice were subjected to swimming exhaustion experiments. Each mouse was first weighed, then a lead wire weighing 5% of the mouse's body mass was tied to its tail. Mice were then placed into a water tank (water depth: 30 cm; temperature: 30°C) and subjected to swimming exhaustion experiments. When mice remained submerged for at least 10 s, they were judged to be exhausted, and the total swimming time till this point was recorded (16).

### Energy Metabolism Index Measurement

Liver and skeletal muscle glycogen was determined using standard methodology (17). Mouse liver was homogenized in 0.5 mL perchloric acid and centrifuged at 24,975 × g for 15 min at 15°C. The resulting supernatant was kept on ice. Thirty microlitres supernatant or glycogen standard was then added to a 96-well microplate, with 200 μL potassium iodide reagent was added subsequently to each well. The absorbance at 460 nm was recorded after standing for 10 min. Serum lactic acid (Lac) content was then determined using the lactate oxidase method (18). Blood urea nitrogen (BUN) content was determined using the diacetyl monooxime color method (19). Serum free fatty acid (NEFA) content was determined using the copper ion color method (20).

### Sports Injury Index Determination

After swimming exhaustion experiments were completed, mice were sacrificed by cervical dislocation, and cardiac blood collected and centrifuged at 4,000 r/min for 20 min. Resulting supernatant serum was retained. Mouse serum CK, AST, and ALT levels were detected using a kit method (Solarbio Life Sciences, Beijing, China).

### Antioxidant Index Determination

SOD and CAT activities and MDA level in mouse serum were measured using detection kits (Solarbio Life Sciences, Beijing, China).

### H&E Staining

For each group, 10 mice undertook swimming exhaustion experiments and the other 5 did not. Mice were sacrificed before and after swimming exhaustion experiments. Mouse livers were washed with normal saline, 0.5 × 0.5 cm^2^ tissue slices were fixed in 10% formalin, embedded in paraffin, and 4 μm thick sections removed. Sections were stained with hematoxylin and eosin (H&E) to evaluate pathological changes in the mouse liver. The resulting staining was observed using an optical microscope (BX43, Olympus, Tokyo, Japan).

### qPCR Assay

Mouse liver tissue and skeletal muscle were collected and homogenized. RNA was then extracted from the tissue with TRIzol™ (Thermo Fisher Scientific, Inc., Waltham, MA, USA) and diluted to 1 μg/μL. One microlitre of the diluted RNA solution was used to obtain a cDNA template using a reverse transcription kit (Solarbio Life Sciences, Beijing, China). One microlitre of the cDNA template was mixed with 10 μL of SYBR Green PCR Master Mix, 1 μL of upstream and downstream primers [see Table 1 (Thermo Fisher Scientific, Inc.)], and 7 μL of sterile distilled water. The mixture was reacted at 95°C for 60 s; then at 95°C for 40 cycles of 15 s each; then at 55°C for 30 s; then at 95°C for 30 s, and finally at 55°C for 35 s. Relative gene expression was calculated using the 2^−ΔΔCt^ method (Stepone Plus qPCR instrument, Thermo Fisher Scientific, Inc.), with GAPDH as the internal reference (21, 22).

**Table 1:** Sequences of the primers used for this experiment.

**Gene name**	**Sequence**
Cu/Zn-SOD	Forward: 5′-AACCAGTTGTGTTGTCAGGAC-3′
	Reverse: 5′-CCACCATGTTTCTTAGAGTGAGG-3′
Mn-SOD	Forward: 5′-CAGACCTGCCTTACGACTATGG-3′
	Reverse: 5′-CTCGGTGGCGTTGAGATTGTT-3′
CAT	Forward: 5′-GGAGGCGGGAACCCAATAG-3′
	Reverse: 5′-GTGTGCCATCTCGTCAGTGAA-3′
Syncytin-1	Forward: 5′-GTTAACTTTGTCTCTTCCAGAATCGA-3′
	Reverse: 5′-CATCAGTACGTGGGCTAGCA-3′
ASCT1	Forward: 5′-ACGCGGGACAGATTTTCAC-3′
	Reverse: 5′-ACACCCGCTGCTCCAAC-3′
iNOS	Forward: 5′-CAAAGGCTGTGAGTCCTGCAC-3′
	Reverse: 5′-ACTTTGATCAGAAGCTGTCCC-3′
TNF-α	Forward: 5′-TGCCACTTCATACCAGGAGA-3′
	Reverse: 5′-CCGGAGTCCGTGATGTCTA-3′
GAPDH	Forward: 5′-GAAGGTGAAGGTCGGAGTCA-3′
	Reverse: 5′-AATGAAGGGGTCATTGATGG-3′

### Statistical Analysis

Excel 2013 software (Microsoft) was used to organize data and draw graphs. Data were expressed as mean ± standard deviation. SPSS18.0 software was used for data analysis. The test methods used were one-way ANOVA and the LSD test. The threshold for statistical significance was set at *p* < 0.05.

## Results

### Swimming Exhaustion Time of Mice

**Figure 1:**
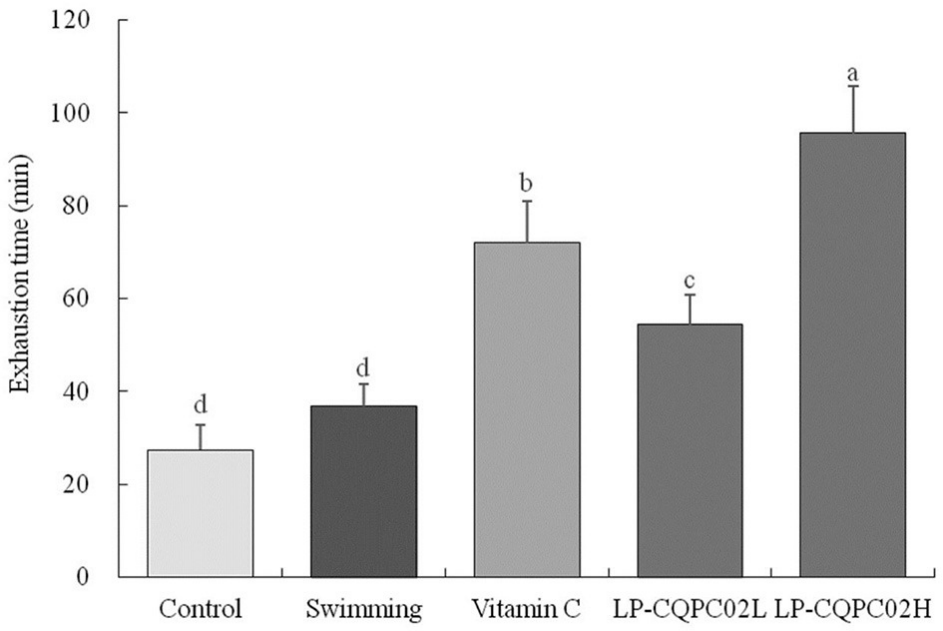
Swimming exhaustion time of mouse in each group. “±” for standard deviation. ^a−d^After the Tukey's honestly significantly different test analysis, there is significant difference between the two groups with different superscript (*P* < 0.05).

Figure 1 shows that the recorded mouse swimming exhaustion time improved significantly after 4 weeks when given either vitamin C (71.92 ± 9.12 min), low dose LP-CQPC02 (54.38 ± 6.32 min), or high dose LP-CQPC02 (95.63 ± 10.02 min) solution by gavage, and that this measured time to exhaustion was significantly higher than that of either the control (27.38 ± 5.36 min) or swimming groups (36.79 ± 4.78 min). Meanwhile, the exhaustion time of the LP-CQPC02H group was significantly higher than that of the vitamin C group. Moreover, the mouse exhaustion time increased with the increasing concentration of LP-CQPC02.

### Energy Metabolism in Mice

Table 2 shows mouse hepatic glycogen, skeletal muscle glycogen and free fatty acid content in the vitamin C, low dose LP-CQPC02, or high dose LP-CQPC02 groups were significantly higher than in control and swimming groups. Hepatic glycogen, skeletal muscle glycogen and free fatty acid content of the LP-CQPC02H group were significantly higher than in the vitamin C group. LP-CQPC02 solution by gavage could increase mouse hepatic glycogen, skeletal muscle glycogen and free fatty acid contents in a dose-dependent fashion. However, mouse lactic acid levels and blood urea nitrogen in the vitamin C and both LP-CQPC02 groups were significantly lower than those seen in the swimming group. Also, lactic acid levels and blood urea nitrogen in the LP-CQPC02H group were significantly lower than those in the vitamin C group. With increasing LP-CQPC02 concentration, lactic acid and blood urea nitrogen levels gradually decrease.

**Table 2:** Hepatic glycogen, skeletal muscle glycogen, lactic acid, blood urea nitrogen and free fatty acid levels of mouse in each group.

**Group**	**Hepatic glycogen**	**Skeletal muscle**	**Lactic acid**	**Blood urea**	**Free fatty**
	**(mg/g)**	**glycogen (mg/g)**	**(mg/L)**	**nitrogen (mg/L)**	**acid (μmol/mL)**
Control	4.03 ± 0.36^e^	1.44 ± 0.32^d^	0.33 ± 0.04^e^	105.36 ± 6.78^e^	420.69 ± 36.78^e^
Swimming	5.12 ± 0.42^d^	2.63 ± 0.39^c^	1.25 ± 0.12^a^	339.75 ± 12.36^a^	638.97 ± 55.79^d^
Vitamin C	9.36 ± 1.02^b^	4.11 ± 0.42^b^	0.78 ± 0.04^c^	212.09 ± 10.50^c^	1147.36 ± 102.32^b^
LP-CQPC2L	7.59 ± 0.77^c^	3.46 ± 0.47^b^^c^	0.96 ± 0.07^b^	265.07 ± 8.56^b^	886.98 ± 88.97^c^
LP-CQPC2H	14.69 ± 1.56^a^	6.86 ± 0.52^a^	0.52 ± 0.06^d^	175.32 ± 12.12^d^	1678.32 ± 154.51^a^

“*±” for standard deviation*.

a−e *After the Tukey's honestly significantly different test analysis, there is significant difference between the two groups with different superscript (P < 0.05)*.

### Sports Injuries in Mice

Serum CK, AST, and ALT levels of mice in the vitamin C and both LP-CQPC02 groups were significantly higher than those in the swimming group. See Table 3. The three indicators in the LP-CQPC02H group were significantly lower than in the vitamin C group. The levels of the three indicators also gradually decreased as the LP-CQPC02 concentration increased.

**Table 3:** Serum CK, AST, and ALT levels of mouse in each group.

**Group**	**CK (U/L)**	**AST (U/L)**	**ALT (U/L)**
Control	72.36 ± 4.56^e^	35.62 ± 3.45^e^	30.52 ± 4.12^e^
Swimming	407.65 ± 38.36^a^	102.78 ± 7.89^a^	95.67 ± 10.92^a^
Vitamin C	225.79 ± 30.32^c^	66.87 ± 6.63^c^	58.78 ± 5.36^c^
LP-CQPC2L	305.87 ± 24.36^b^	78.32 ± 4.85^b^	71.08 ± 6.11^b^
LP-CQPC2H	145.97 ± 23.75^d^	50.23 ± 5.63^d^	42.06 ± 3.60^d^

“*±” for standard deviation*.

a−e *After the Tukey's honestly significantly different test analysis, there is significant difference between the two groups with different superscript (P < 0.05)*.

### Serum Oxidation Level of Mice

Table 4 shows serum SOD and CAT levels in the vitamin C and both LP-CQPC02 groups were significantly higher than in the swimming group. Moreover, supplementation of LP-CQPC02 increases SOD and CAT levels in a dose-dependent manner. MDA levels in the vitamin C and both LP-CQPC02 groups were significantly lower than in the swimming group. Again, supplementation of LP-CQPC02 lowered MDA levels in a dose-dependent manner.

**Table 4:** Serum SOD, CAT, and MDA levels of mouse in each group.

**Group**	**SOD (U/L)**	**CAT (U/L)**	**MDA (μmol/L)**
Control	52.36 ± 4.36^e^	32.58 ± 2.98^e^	4.56 ± 0.42^d^
Swimming	68.97 ± 4.77^d^	45.78 ± 4.02^d^	15.76 ± 0.56^a^
Vitamin C	112.36 ± 9.24^b^	75.63 ± 5.62^b^	8.12 ± 0.66^c^
LP-CQPC2L	87.92 ± 7.98^c^	60.32 ± 4.88^c^	10.26 ± 0.51^b^
LP-CQPC2H	163.05 ± 12.12^a^	122.47 ± 9.16^a^	5.39 ± 0.57^cd^

“*±” for standard deviation*.

a−e *After the Tukey's honestly significantly different test analysis, there is significant difference between the two groups with different superscript (P < 0.05)*.

### Pathological Observation

**Figure 2:**
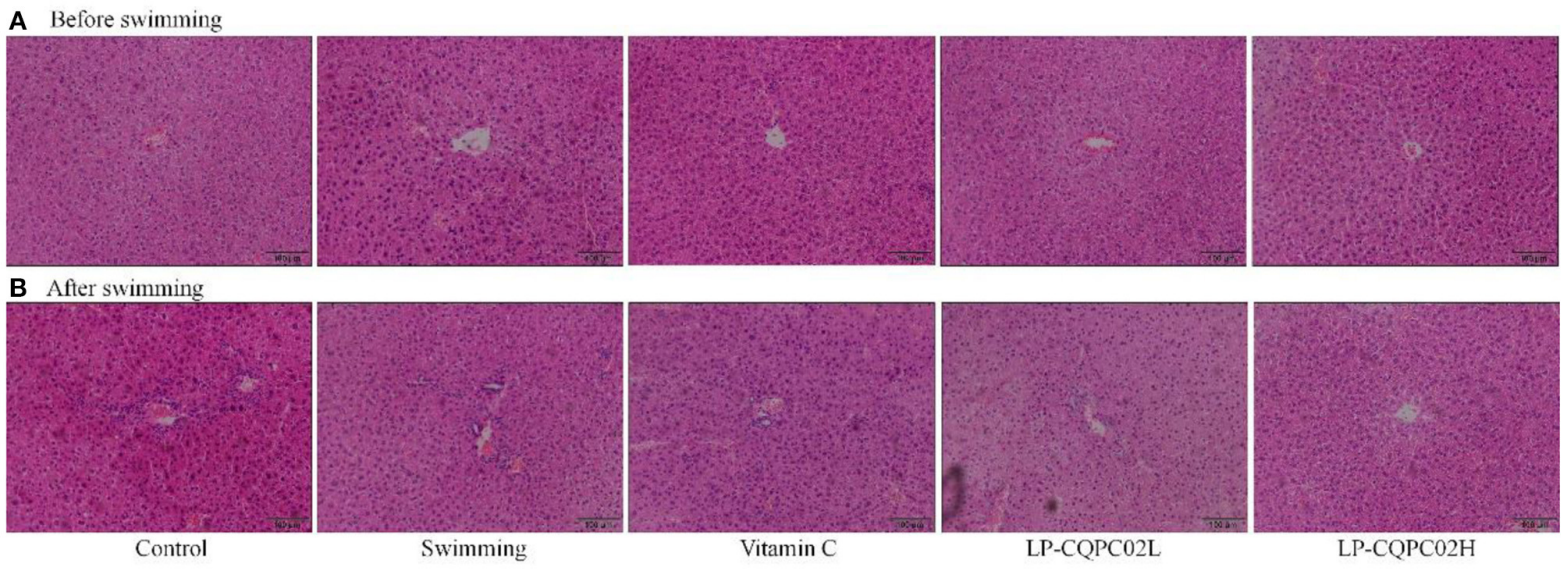
Pathological observation of hepatic tissue H&E section of **(A)** before swimming and **(B)** after swimming mouse in each group.

Before the swimming exhaustion experiment, mouse liver nuclei from each group were uniformly stained. See Figure 2A. The structure of mouse liver cells was normal, with hepatocytes radially distributed around the central vein. This indicated that the vitamin C and LP-CQPC02 groups had no obvious pathological effects on mouse liver. After the swimming experiment, mouse cells from each group (Figure 2B) appeared unevenly arranged, with the central vein appearing irregular, and some cell structures were destroyed or necrotic. Both vitamin C and LP-CQPC02 alleviated the liver damage caused by exhaustion swimming, with LP-CQPC02H having the best effect.

### Expression of Cu/Zn-SOD, Mn-SOD, and CAT mRNA in Mouse Liver

**Figure 3:**
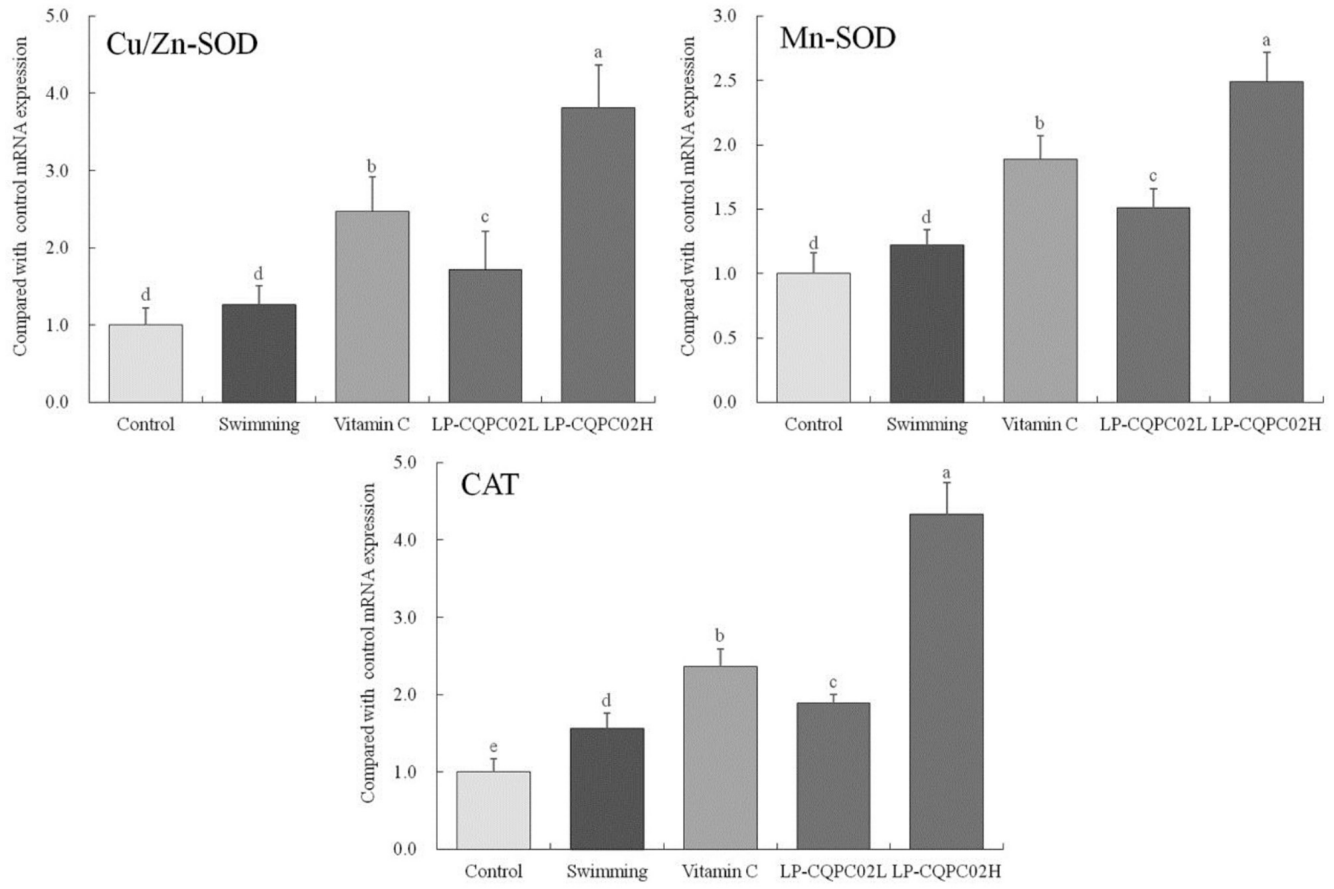
Cu/Zn-SOD, Mn-SOD, and CAT mRNA expression of hepatic tissue in mouse. ^a−e^After the Tukey's honestly significantly different test analysis, there is significant difference between the two groups with different superscript (*P* < 0.05).

Figure 3 shows that mRNA expression of Cu/Zn-SOD, Mn-SOD, and CAT was weakest in control mice (1.00 ± 0.22, 1.00 ± 16, and 0.17 folds of control), while their expression in the swimming group mice (1.26 ± 0.25, 1.22 ± 0.12, and 1.25 ± 0.20 folds of control) was increased compared to the control group. Expression in vitamin C (2.47 ± 0.45, 1.89 ± 0.18, and 2.36 ± 0.23 folds of control), LP-CQPC02L (1.72 ± 0.49, 1.51 ± 0.15, and 1.89 ± 0.11 folds of control), and LP-CQPC02H (3.81 ± 0.56, 2.49 ± 0.23, and 4.33 ± 0.41 folds of control) groups was significantly up-regulated when compared to the control and swimming groups, with expression in the LP-CQPC02H group the strongest.

### The mRNA Expression of Syncytin-1, ASCT1, iNOS and TNF-α in Mouse Skeletal Muscle

**Figure 4:**
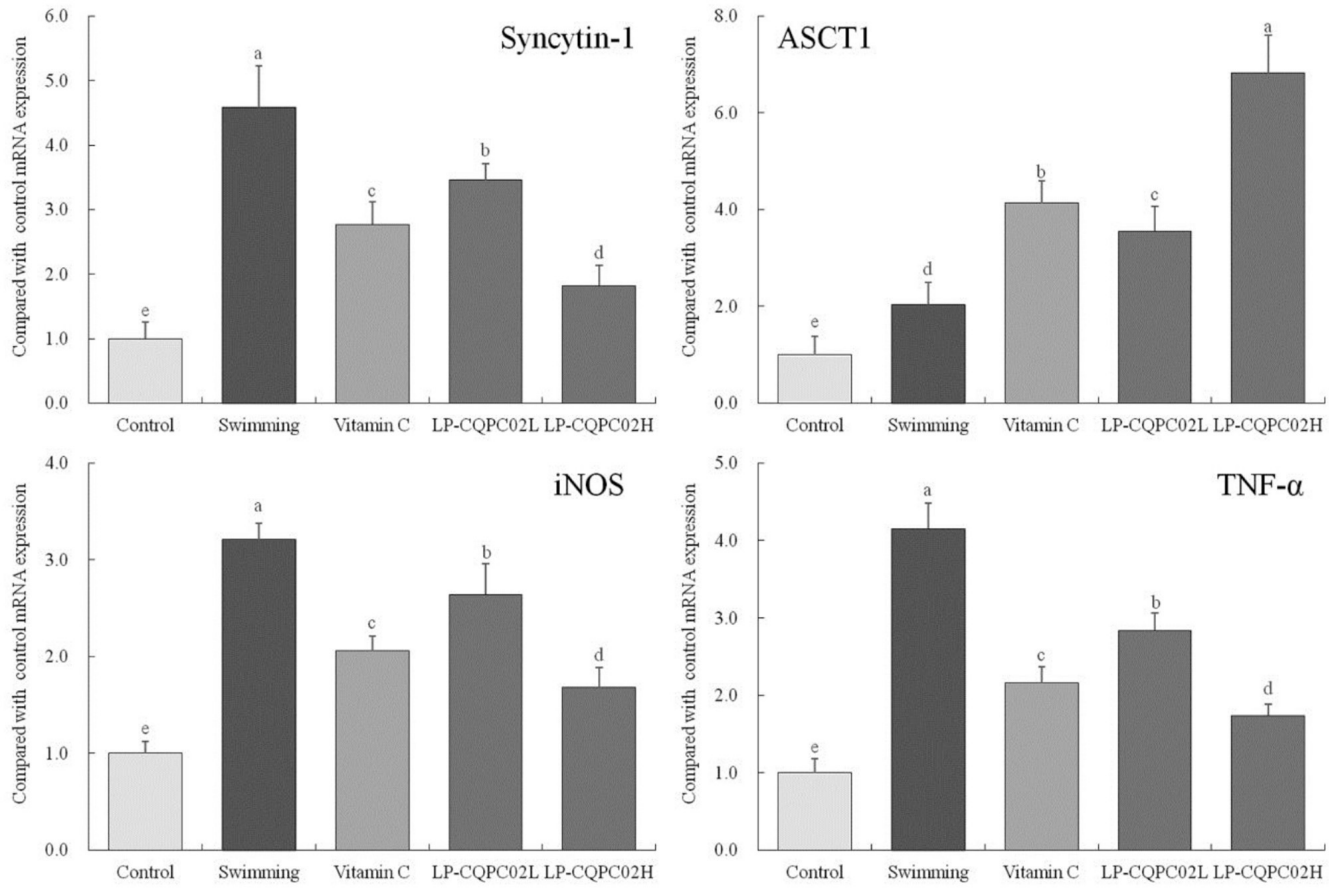
Syncytin-1, ASCT1, iNOS and TNF-α mRNA expression of skeletal muscle c tissue in mouse. ^a−e^After the Tukey's honestly significantly different test analysis, there is significant difference between the two groups with different superscript (*P* < 0.05).

Figure 4 shows the mRNA expression of syncytin-1 (1.00 ± 0.25, 1.82 ± 0.31, 2.77 ± 0.35, 3.45 ± 0.25, and 4.58 ± 0.65 folds of control), iNOS (1.00 ± 0.12, 1.68 ± 0.21, 2.06 ± 0.15, 2.64 ± 0.32, and 3.2 ± 0.17 folds of control), and TNF-α (1.00 ± 0.18, 1.74 ± 0.15, 2.16 ± 0.21, 2.84 ± 0.22 and 4.15 ± 0.33 folds of control) in mouse skeletal muscle from each group were, in descending order: control, LP-CQPC02H, vitamin C, LP-CQPC02L, and swimming. The difference was that ASCT1 expression ascending through the groups: control, then swimming, then LP-CQPC02L, vitamin C, and finally LP-CQPC02H groups (1.00 ± 0.38, 2.03 ± 0.46, 3.55 ± 0.52, 4.13 ± 0.46 and 6.82 ± 0.78 folds of control).

## Discussion

The mechanism underlying exercise fatigue comprises failures in energy supply, metabolite accumulation, and free radical attack (23). Energy supply failure is due to the excessive consumption of stored long- and short-term energy substances, such as glycogen and glucose, and reduced dynamic supply due to excessive or intense exercise (24). Metabolite accumulation can be affected by strenuous or excessive exercise, leading aerobic energy supply to convert to anaerobic glycolysis, thus, the energy contained in glucose (or glycogen) is used to form adenosine triphosphate (ATP), muscle uses the anaerobic decomposition of ATP and creatine phosphate (CP) to produce lactic acid and release energy. The metabolites of this process, including lactic acid, ammonia, and other anaerobic fermentation products accumulate in significant amounts, perturbing acid-base homeostasis and causing imbalances in the intracellular microenvironment, thus inducing fatigue (25). Free radical attack is due to anaerobic fermentation increasing levels of oxygen free radicals, which destroy the fluidity and permeability of cell membranes, inducing tissue damage, and thus cause fatigue (26).

Pathological fatigue may require drug treatment. Many with weakened resistance or other conditions are prone to exercise fatigue when undertaking mild exercise. Drug interventions often have side effects, and for these populations the alleviation of fatigue often includes physiotherapy, nutritional supplements, and biologics (27). Nutritional supplements and biological preparations are the principal areas of current fatigue research. Lactic acid bacteria have been the subject of many current studies, it can improve immunity, preventing cardiovascular diseases, mediate anti-aging, act as antioxidant, and thus generate anti-fatigue effects (28–30). There are studies that indicate mechanisms of actions, the lactic acid bacteria could possibly improve exercise performance via energy regulation and muscular adaptation (31, 32). The results also verified the fatigue resistance of a new lactic acid bacteria (LP-CQPC02), and proved that the probiotic potential lactic acid bacteria had this effect.

The exhaustion swimming experiment is a commonly used *in vivo* mouse model of human fatigue used to observe the effects of research interventions on exercise ability and fatigue mitigation (33). In this study, the anti-fatigue effect and antioxidant capacity of lactic acid bacteria LP-CQPC02 were investigated using this test, with vitamin C used as a positive control. Different doses of LP-CQPC02 could significantly increase the swimming exhaustion time in mice. Moreover, the time to exhaustion of LP-CQPC02 was significantly greater than that of vitamin C at the same nominal dose. Mouse exhaustion time was prolonged by increasing concentrations of LP-CQPC02, indicating that LP-CQPC02 had a more pronounced anti-fatigue effect.

Liver glycogen, skeletal muscle glycogen, lactic acid, blood urea nitrogen and free fatty acids are all important in energy supply (34). Hepatic glycogen is polymerized glucose molecules stored in the liver. When the body requires energy, hepatic glycogen is hydrolyzed to glucose liberating energy. Therefore, hepatic glycogen and skeletal muscle can be a key indicator of fatigue. Free fatty acids are the breakdown products of fat. Vigorous or extended exercise mobilizes fat, increasing available free fatty acids, which provides energy for the body and alleviates fatigue (35). We found that after exhaustive exercise, hepatic glycogen, skeletal muscle glycogen and free fatty acid levels in mice from the vitamin C and LP-CQPC02 groups were significantly higher than those from the control and swimming groups. Hepatic glycogen, skeletal muscle glycogen and free fatty acids in the LP-CQPC02H group were significantly higher than those in the vitamin C group. Supplementation of LP-CQPC02 could raise mouse hepatic glycogen, skeletal muscle glycogen and free fatty acid levels in a dose-dependent manner. Meanwhile, because glycogen stores in the body are not very large and the general anaerobic exercise duration is short (25). These observations suggest that LP-CQPC02 can enhance hepatic glycogen, skeletal muscle glycogen reserves and thereby allowing the anaerobic exercise time to grow and prolong the time when fatigue occurs, which plays an anti fatigue role.

Vigorous or long-term exercise can induce a shift to anaerobic glycolysis, resulting in the metabolic production of lactic acid, reducing the pH of the internal environment, reducing the contractile capacity of muscles, thus inducing fatigue (36). Moreover, strenuous or long-term exercise can also cause protein degradation producing urea, which is the most obvious manifestation of protein catabolism in the body (37). We found that after exercise, mouse lactic acid and blood urea nitrogen levels in the vitamin C and LP-CQPC02 groups were significantly lower than those of the swimming group. Likewise, lactic acid and blood urea nitrogen levels in the LP-CQPC02H group were significantly lower than those in the vitamin C group. As LP-CQPC02 concentration rose, lactic acid and blood urea nitrogen levels gradually decreased. This suggests LP-CQPC02 supplementation may help reduce lactic acid accumulation and inhibit protein degradation, and thus alleviate fatigue through positive regulation of energy metabolism.

During exercise, muscle and liver are the most vulnerable sites to injury. Such injury is usually accompanied by increased CK, AST, and ALT levels (38). We found that serum CK, AST, and ALT levels in mice in vitamin C and LP-CQPC02 groups were significantly higher than those in the swimming group, and these three indicators were significantly lower in the LP-CQPC02H group than in the vitamin C group. These three levels also gradually decreased with increasing LP-CQPC02 concentration. Thus, we concluded that LP-CQPC02 functions to prevent motor injury during fatigue-inducing exercise.

Excessive exercise can promote the creation and accumulation of superoxide free radicals, which can damage cell membranes and cellular metabolism, causing oxidative stress in tissues and organs. SOD and CAT are important antioxidant enzymes: SOD can effect repair to damaged cells as well as its antioxidant effects. In vertebrates SOD is expressed primarily as Cu/Zn-SOD and Mn-SOD (39). SOD converts free radicals into less toxic H_2_O_2_. These are in turn converted to H_2_O by CAT, thereby scavenging free radicals (40). We found that both the vitamin C and LP-CQPC02 groups had significantly higher SOD and CAT levels than the swimming group. Supplementation of LP-CQPC02 could increase SOD and CAT levels in a dose-dependent manner. This suggests that LP-CQPC02 has good antioxidant capacity. MDA is the oxidation product of cell membrane lipids and is a sensitive index of lipid peroxidation. During fatigue, MDA levels typically increase (41). In this study, MDA levels in the vitamin C and LP-CQPC02 groups were significantly lower than in the swimming group. Supplementation of LP-CQPC02 reduced MDA levels in a dose-dependent manner, suggesting LP-CQPC02 may have a protective and antioxidant role through attenuating lipid peroxidation.

Studies indicate syncytin-1 expression is enhanced in muscle tissues after injury, inflammation and atrophy, and thus affects the capacity for exercise (42). This enhanced expression of syncytin-1 leads to inhibited expression of its receptor ASCT1, and decreased ASCT1 expression, which is regulated by nitric oxide (NO) (43). iNOS levels increase in response to inflammation, resulting in high levels of NO production, which in turn inhibits ASCT1 expression (44). However, TNF-α can induce and increase syncytin-1 expression in muscle, causing production of free radicals and cyto-inflammatory factors, which also leads to inhibited ASCT1 expression (45). Our study showed that vitamin C and LP-CQPC02 could up-regulate ASCT1 expression and down-regulate syncytin-1, iNOS, TNF-α expression in mouse skeletal muscle after exhaustive exercise. The same results were obtained with previous studies. It can be seen that LP-CQPC02 can alleviate the muscle tissue damage caused by exhaustive exercise.

A weight of evidence supports lactic acid bacteria improving the composition of intestinal flora, promoting digestion and absorption, and enhancing immune function (46). As the beneficial health effects of lactic acid bacteria are numerous, studies have confirmed that certain lactic acid bacteria can reduce fatigue, although specific mechanistic details remain poorly studied (47). Some studies show that lactic acid bacteria have good antioxidant effects *in vitro* and *in vivo* (48). Our study has confirmed LP-CQPC02 to have a good antioxidant effect *in vivo*, producing anti fatigue effects. This study has elucidated part of the mechanism, but deeper mechanistic investigations are needed in the future.

## Conclusions

In summary, LP-CQPC02 has excellent anti-fatigue and antioxidant properties, and its anti-fatigue effect is related to improvements in hepatic glycogen, skeletal muscle glycogen reserve capacity, increasing fat mobilization, reducing lactic acid accumulation and protein decomposition. Its antioxidant capabilities are related to scavenging free radicals and reducing lipid peroxidation. In conclusion, LP-CQPC02 has great and untapped potential as a microbial drug preparation or nutritional supplement for exercise.

## Data Availability Statement

The original contributions presented in the study are included in the article/supplementary material, further inquiries can be directed to the corresponding author/s.

## Ethics Statement

The protocol for the experiments performed in this animal study was reviewed and approved by the Ethics Committee of Chongqing Collaborative Innovation Center for Functional Food (202005022B), Chongqing, China.

## Author Contributions

RY and MF performed the majority of the experiments and wrote the manuscript. QC and XL contributed to the data analysis. K-YP and XZ designed and supervised the study and checked the final manuscript. All authors contributed to the article and approved the submitted version.

## Conflict of Interest

The authors declare that the research was conducted in the absence of any commercial or financial relationships that could be construed as a potential conflict of interest.
